# MicroRNAs and Their Influence on the ZEB Family: Mechanistic Aspects and Therapeutic Applications in Cancer Therapy

**DOI:** 10.3390/biom10071040

**Published:** 2020-07-12

**Authors:** Milad Ashrafizadeh, Hui Li Ang, Ebrahim Rahmani Moghadam, Shima Mohammadi, Vahideh Zarrin, Kiavash Hushmandi, Saeed Samarghandian, Ali Zarrabi, Masoud Najafi, Reza Mohammadinejad, Alan Prem Kumar

**Affiliations:** 1 Department of Basic Science, Faculty of Veterinary Medicine, University of Tabriz, Tabriz 5166616471, Iran; dvm.milad73@yahoo.com; 2 Department of Pharmacology, Yong Loo Lin School of Medicine, National University of Singapore, Singapore 117600, Singapore; e0336095@u.nus.edu; 3 Cancer Science Institute of Singapore, National University of Singapore, Singapore 117599, Singapore; 4 Department of Anatomical Sciences, School of Medicine, Student Research Committee, Shiraz University of Medical Sciences, Shiraz 7134814336, Iran; Ebrahimrahmani1374@gmail.com; 5 General Practitioner, Kerman University of Medical Sciences, Kerman 7616913555, Iran; Shima.mohamadi92@yahoo.com; 6 Laboratory for Stem Cell Research, Shiraz University of Medical Sciences, Shiraz 7134814336, Iran; zarrin.vahideh2075@gmail.com; 7 Department of Food Hygiene and Quality Control, Division of Epidemiology & Zoonoses, Faculty of Veterinary Medicine, University of Tehran, Tehran 1417414418, Iran; houshmandi.kia7@ut.ac.ir; 8 Department of Basic Medical Sciences, Neyshabur University of Medical Sciences, Neyshabur 9318614139, Iran; samarghandians@mums.ac.ir; 9 Sabanci University Nanotechnology Research and Application Center (SUNUM), Tuzla 34956, Istanbul, Turkey; alizarrabi@sabanciuniv.edu; 10 Center of Excellence for Functional Surfaces and Interfaces (EFSUN), Faculty of Engineering and Natural Sciences, Sabanci University, Tuzia, Istanbul 34956, Turkey; 11 Radiology and Nuclear Medicine Department, School of Paramedical Sciences, Kermanshah University of Medical Sciences, Kermanshah 6715847141, Iran; najafi_ma@yahoo.com; 12 Neuroscience Research Center, Institute of Neuropharmacology, Kerman University of Medical Sciences, Kerman 7619813159, Iran

**Keywords:** microRNA, ZEB family, cancer therapy, EMT, drug resistance, immunotherapy, non-coding RNA

## Abstract

Molecular signaling pathways involved in cancer have been intensively studied due to their crucial role in cancer cell growth and dissemination. Among them, zinc finger E-box binding homeobox-1 (ZEB1) and -2 (ZEB2) are molecules that play vital roles in signaling pathways to ensure the survival of tumor cells, particularly through enhancing cell proliferation, promoting cell migration and invasion, and triggering drug resistance. Importantly, ZEB proteins are regulated by microRNAs (miRs). In this review, we demonstrate the impact that miRs have on cancer therapy, through their targeting of ZEB proteins. MiRs are able to act as onco-suppressor factors and inhibit the malignancy of tumor cells through ZEB1/2 down-regulation. This can lead to an inhibition of epithelial-mesenchymal transition (EMT) mechanism, therefore reducing metastasis. Additionally, miRs are able to inhibit ZEB1/2-mediated drug resistance and immunosuppression. Additionally, we explore the upstream modulators of miRs such as long non-coding RNAs (lncRNAs) and circular RNAs (circRNAs), as these regulators can influence the inhibitory effect of miRs on ZEB proteins and cancer progression.

## 1. Introduction

Epithelial-mesenchymal transition (EMT) process was first introduced by Greenburg and his colleagues in 1982 [1]. To date, three major types of EMT have been identified: type I EMT, which occurs during embryogenesis, type II EMT, which is activated during wound healing, tissue regeneration and organ fibrosis, and type III EMT, which occurs during metastasis of cancer cells [2]. EMT is the process of cellular transition wherein epithelial cells are bio-transformed into mesenchymal cells with fibroblast-like properties [3,4,5,6]. In the EMT mechanism, cadherins play a significant role. Cadherins promote cell-cell adhesion and are located at the adherens’ junctions. There are different kinds of cadherins including E, N, P, VE, proto, desmosomal, and FAT cadherins, but N-cadherin and E-cadherin are the most important ones in EMT mechanism. A decrease in E-cadherin levels, and an increase in N-cadherin levels lead to stimulation of EMT, and enhanced migratory ability of cancer cells [7,8]. Additionally, upon EMT stimulation, morphology changes and alterations in cytoskeleton occur in cells and affect their migratory ability and adhesion to neighboring cells. These molecular and structural changes promote the dissemination of cells into other sites [9]. Essentially, this increased cell migration is beneficial in normal cells to accelerate physiological processes such as wound healing and embryogenesis. It has been reported that EMT occurs to provide the required flexibility for mesoderm and neural crest formations [10,11]. However, cancer cells can exploit the EMT mechanism for metastasis to distant sites [12,13,14]. There is increased attention towards the EMT mechanism in cancer therapy not only because of its contribution toward metastasis, but also due to the fact that the EMT mechanism can trigger chemoresistance of cancer cells, and decrease sensitivity to apoptosis [15,16]. Therefore, understanding the molecular pathways regulating EMT is a crucial in the field of cancer studies.

EMT is regulated by a variety EMT-promoting transcription factors (EMT-TFs) such as Snail, Slug, Twist, TBX-2, SIX, transforming growth factor--β (TGF-β), and Zinc finger E-box-binding homeobox protein (ZEB) [17]. These upstream EMT-TFs can induce EMT and promote the biotransformation of cells from epithelial phenotype into mesenchymal phenotype by affecting levels of cadherins. Different studies have shown the involvement of ZEB proteins in modulating EMT during normal development and in pathological conditions [18,19,20,21]. Our aim in the present review is to 1) show that ZEB proteins are able to regulate metastasis of cancer cells via affecting EMT, 2) understand how different microRNAs (miRs) can regulate the ZEB/EMT axis, and 3) demonstrate how other upstream mediators can regulate the miR/ZEB/EMT axis.

## 2. ZEB Family

The ZEB family, which was first discovered in Drosophila melanogaster, consists of two key members ZEB1 and ZEB2 [22]. Both ZEB1 and ZEB2 possess the amino-terminal (NZF) and carboxy-terminal zinc finger cluster (CZF), thereby allowing them to bind to regulatory DNA sequences in their target promoters [23,24,25]. This has led to their involvement in different biological events, such as embryogenesis, hematopoiesis, and more importantly, EMT. In fact, ZEB proteins are well-known due to their ability in stimulation of EMT [20]. In this section, we provide an overview of ZEB1 and ZEB2 proteins to shed some light on their role in cancer cells.

### 2.1. ZEB1

ZEB1 gene is located on chromosome 10p11.2, and its protein is made up of two zinc-finger clusters at N- and C-terminal ends, while the middle portion of the ZEB1 protein contains three distinct parts including a homeodomain, a Smad interaction domain and a C-terminal binding protein (CtBP). The CtBP is involved in the regulation of ZEB1 function [26,27]. Primarily, the zinc-finger clusters allow ZEB1 to bind to E-boxes. ZEB1 regulates its downstream effectors through binding to E-promoter DNA sequence (5′-CANNTG-3′) [28]. Various publications have also highlighted ZEB1′s association with enhanced viability and invasiveness of cancer cells. In colorectal cancer (CRC) cells, it was found that tumor suppressor death domain-associated protein (DAXX) is able to prevent ZEB1 modulation on E-cadherin to inhibit the invasion and proliferation of tumor cells. Down-regulation of DAXX enhanced ZEB-1 suppression of E-cadherin, leading to the enhanced proliferation and malignancy of cancer cells [29]. It has also been highlighted that EMT may contribute to chemoresistance of cancer cells [30,31]. In pancreatic cancer, Rho associated coiled coil containing protein kinase 2 (ROCK2) enhances the expression of ZEB1. This in turn leads to ZEB1-mediated EMT induction, which contributes to gemcitabine resistance in pancreatic cancer cells [32]. In CRC cells, TCF4 enhances expression of ZEB1 to promote stemness and migration of cancer cells, thereby promoting chemotherapy resistance [33]. In prostate cancer cells, ZEB1 stimulates up-regulation of ATP-binding cassette subfamily C member 10 (MRP4) to export docetaxel out of cancer cells, resulting in their decreased sensitivity to chemotherapy [34]. These studies support the modulation of ZEB1, and highlights that it may be beneficial in enhancing the efficacy of chemotherapy and in reducing the migratory ability of cancer cells. Overall, ZEB1 is an important mediator to enhance the invasion and proliferation of tumor cells. More importantly, ZEB1 may significantly reduce the efficiency of chemotherapy.

### 2.2. ZEB2

ZEB2 is another member of ZEB family and is located on chromosome 2q22.3 [25]. Structurally, the N-terminal end of ZEB2 consists of four zinc fingers, while the C-terminal end has three zinc fingers [25]. Similar to ZEB1, ZEB2 appears to play a crucial role in migration and invasion. In non-small cell lung cancer (NSCLC), MDM2 binding protein (MTBP) behaves as an oncogene to increase EMT through ZEB2 up-regulation [35]. This in turn enhanced the migration and metastasis of NSCLC tumor cells. In bladder cancer, it was found that indoleamine-2,3-dioxygenase-1 (IDO1) induces ZEB2 overexpression, which in turns increases the viability and proliferation of cancer cells [36]. ZEB2 has also been found to increase the expression of ETS proto-oncogene 1 (ETS1) to up-regulate other EMT proteins such as matrix metalloproteinase 9 (MMP-9) and Twist [37]. Importantly, ZEB2 is also capable of inducing chemoresistance via EMT activation. Phosphatidylinositol 3-kinase (PI3K)/protein kinase-B (Akt) pathway is a down-stream pathway of ZEB2 that induces EMT by reducing the level of E-cadherin protein, leading to the generation of cisplatin resistance in NSCLC cells [38] In all, ZEB2 appears to mediate EMT, and may be a potential therapeutic target in cancer treatment.

## 3. MicroRNAs

Non-coding RNAs (ncRNAs) comprise a huge part of human genome and are involved in various molecular pathways and processes [39,40,41,42,43,44,45,46,47,48]. They are divided into two characteristic groups: house-keeping and regulatory molecules [49]. They play a remarkable role in vital biological processes such as apoptosis, autophagy, differentiation, cell cycle, proliferation, and migration by targeting various down-stream molecular pathways [50,51]. Additionally, they contribute to the transcription, post-transcriptional modifications, and signal transduction networking. MiRs are house-keeping molecules belonging to the small nucleolar RNAs (SnoRNAs) family [52,53,54]. In this section, we first provide an introduction about miRs and their biosynthesis, followed by a highlight of their potential roles in cancer.

MiRs are single-stranded RNA molecules with a length of 19–24 nucleotides and may possess regulatory functions [55,56]. In total, 60% of all human genome has a binding site for miRs. This highlights the influence of miRs as they control many cellular processes and their dysregulation is related to the development of diseases [57,58,59,60,61]. MiRs are capable of post-transcriptional regulation of their target through RNA interference. These small RNA molecules bind to their target via 3′-untranslated region (3′-UTR). It has been demonstrated that the level of miRs has a negative relationship with the expression of their down-stream targets [62,63,64]. Moreover, one miR is able to target more than one messenger RNA (mRNA), again highlighting their widespread influence in many cellular processes [65]. In the synthesis of miR, a primary miR (pri-miR) is first produced by the action of RNA polymerase. The pri-miR is long with more than 500 nucleotides. It is then processed by Drosha/Pasha and DICER1 proteins, which cleave the pri-miR to generate a mature miR. Next, the mature miR is incorporated in a complex to form miR-RNA-induced silencing complex assembly [66,67,68,69,70].

### MicroRNAs in Cancer Metastasis

When focusing on the cancer context, miRs can have oncogenic, or tumor suppressing properties. Onco-suppressor miRs that inhibit invasion of cancer cells undergo down-regulation during cancer development. Enhancing the expression of such miRs can aid in the down-regulation of factors involved in migration of cancer cells such as PRMT5 [71]. Additionally, MiR-506-3p up-regulation considerably reduces the viability and proliferation of ovarian cancer cells and stimulates apoptotic cell death. Investigation of underlying molecular pathways shows that miR-506-3p inhibits Akt/Forkhead box O3 (FOXO3a) by inhibition of sirtuin 1 (SIRT1) [72]. Elevating the expression of miR-506-3p is a potential strategy in ovarian cancer treatment. In the gastric cancer model, it was also observed that a reverse relationship between miR-612 and nin one binding protein (NOB1) helped reduce the migration and invasion of cervical cancer cells [73]. Similarly, in pancreatic cancer, it was also found that overexpression miRs could lead to better prognosis. Enhancing the expression of miR-519 appears to sensitize pancreatic cancer cells to apoptosis and inhibits their proliferation and migration. This miR prevents the activation of programmed death ligand 1 (PD-L1), under hypoxic conditions to suppress tumorigenesis [74]. In all, various studies have shown that miRs are efficient upstream mediators that target various molecular pathways. Enhancing the expression of tumor suppressor miRs may prove to be an advantageous strategy and extensive research is currently being performed to exploit this strategy [74,75,76,77,78]. Conversely, oncogenic miRs are able to elevate the malignancy and proliferation of cancer cells and are associated with poor prognosis. Their downregulation is of interest in cancer therapy [79,80]. For instance, miR-424-5p is able to induce anoikis resistance to promote migratory ability of cancer cells [81]. The targeting of miRs may therefore be considered a promising candidate in cancer therapy. Interestingly, EMT-TFs are considered as potential down-stream targets of miRs in cancer metastasis. MiR-582-3p and miR-582-5p suppress migration of cancer cells via down-regulation of TGF-β in cancer cells [82]. This concurs that miRs can play a significant role in the regulation of metastasis via targeting different pathways and mechanisms. In the following sections, we focus on the regulation of ZEB proteins by miRs and their association with cancer metastasis and chemoresistance.

## 4. MicroRNA, ncRNA, and ZEB: Role in EMT and Cancer Metastasis

This section specifically demonstrates the impact that miRs have on cell migration and invasion, through their targeting of ZEB proteins. Upstream modulators of miRs such as lncRNAs and circRNAs are also extensively discussed. As mentioned, miRs are able to act as both onco-suppressor as well as promoter of cancer dissemination. Particularly, they are able to exert these effects through their modulation of ZEB proteins, to result in changes in the EMT mechanism. For instance, it appears that miR-200c plays a dual role in cancer therapy. Some studies have demonstrated that miR-200c elevates the viability and proliferation of tumor cells, while another study showed that miR-200c sensitizes cancer cells into chemotherapy by targeting neurophilin 1 and reducing cancer malignancy [83,84,85,86]. It is believed that miR-200c exerts an inhibitory impact on TGF-β-mediated EMT through down-regulation of both ZEB1 and ZEB2 proteins [87].

### 4.1. ZEB1

#### 4.1.1. MiRs as Modulators of ZEB1

Breast cancer is one of the leading causes of death among females [88]. Importantly, metastasis is a prevalent concern in breast cancer development [89]. Notably, ZEB1 has been identified as a key player in migration of breast cancer cells. MiR-200a was found to down-regulate ZEB1 in suppressing cancer cell migration. It appears that by down-regulating ZEB1, miR-200a can enhance E-cadherin levels and inhibit EMT [90]. This study demonstrates that the relationship between miRs and ZEB proteins is crucial in the regulation of metastasis. Additionally, other onco-suppressor miRs have also been identified to regulate the migration of cancer cells. MiR-1271 have been found to significantly decrease the viability and proliferation of tumor cells [91,92,93]. In ovarian cancer cells, miR-1271 inhibits EMT via ZEB1 down-regulation (binding into 3′-UTR), leading to the decreased viability, proliferation, invasion, and migration of tumor cells. As a consequence of ZEB1 down-regulation by miR-1271, levels of E-cadherin undergo up-regulation, accompanied by a decrease in the levels of N-cadherin [94]. In gastric cancer cells, expression of miR-203 undergoes down-regulation, resulting in an up-regulation of ZEB1 and resistance of cancer cells to radiotherapy. It has been suggested that enhancing the expression of miR-203 is a potential strategy in sensitizing cancer cells to radiotherapy, since miR-203 binds to the 3′-UTR of ZEB1 to repress its expression. This then results in a decrease in the malignancy of cancer cells and an increased sensitivity to radiotherapy [95]. Additionally, inhibition of ZEB1 by miRs such as miR-101-3p, miR-525-5p and miR-186-5p is also corelated with a diminution in metastasis of cancer cells due to EMT inhibition by E-cadherin up-regulation [96,97,98]. Taken together, the miR/ZEB1 axis is an important factor in cancer dissemination and may be an important and relevant target in cancer therapeutics. A newly published study has investigated efficacy of ursolic acid in affecting miR-220c/ZEB1 axis. Ursolic acid enhances expression of miR-200c, as an onco-suppressor factor that, in turn, reduces expression of TGF-β1, providing the condition for down-regulation of ZEB1 and inhibiting metastasis of CRC cells [97].

As aforementioned, EMT-TFs such as TGF-β can stimulate EMT. ZEB1 engages in a feedback loop with TGF-β and miR, thereby promoting metastasis of cancer cells. Normally, miR-33a-5p suppresses TGF-β to inhibit ZEB1 activation, leading to suppression of metastasis. However, in cancer conditions, TGF-β and ZEB1 cooperate with each other to promote migration of cancer cells. TGF-β can enhance copy numbers of ZEB1, while ZEB1 suppresses miR-33a-5p, an inhibitor of TGF-β signaling. This cooperation between ZEB1 and TGF-β leads to inhibition of miR-33a-5p, and stimulation of EMT [99]. This once again highlights that onco-suppressor miRs may suppress ZEB1 via affecting other EMT-TFs such as TGF-β, and that ZEB1 can form a negative feedback loop with onco-suppressor miRs in promoting metastasis of cancer cells.

The Akt/mammalian target of rapamycin (mTOR) signaling pathway is another pathway that is commonly deregulated in cancer [100,101,102]. Phosphorylated Akt can induce mTOR to promote the motility and invasion of tumor cells [103,104,105,106,107,108]. It appears that miR-205 is able to target the Akt/mTOR signaling pathway to regulate malignancy and progression of cancer cells [109]. By suppressing Akt/mTOR signaling pathway, miR-708 acts as an anti-tumor agent to inhibit ZEB1, leading to the suppressing EMT mechanism [110]. Finally, miR-126 was also found to inhibit ZEB1 to suppress MMP-2, MMP-9, and oncogenic JAK2/STAT3 signaling pathway, leading to the reduced migration and metastasis of cervical cancer cells [111].

Additionally, Wnt signaling pathway contributes to cancer cell growth and dissemination. Abnormal expression of Wnt signaling pathway can be observed in cancers [112,113,114,115,116,117]. Wnt/β-catenin signaling pathway can promote EMT through ZEB1 up-regulation to elevate the invasion and malignancy of tumor cells. Enhancing the expression of miR-33b effectively inhibits Wnt/β-catenin/ZEB1 axis to suppress cancer malignancy through EMT inhibition [118]. Similarly, miR-200a is capable of decreasing gastric adenocarcinoma invasion via down-regulation of Wnt/β-catenin and subsequent suppressing of ZEB1 and ZEB2 [119]. In malignant meningioma, miR-4652-3p down-regulates the expression of ZEB1 by suppressing Wnt and nuclear translocation of β-catenin. Conversely, lncRNA LINC00702 can activate Wnt/β-catenin signaling pathway by sponging miR-4652-3p to induce ZEB1 and promote the metastasis and invasion of malignant meningioma [120]. These studies highlight the fact that firstly, Wnt can promote metastasis of cancer cells via ZEB1 up-regulation; secondly, the Wnt/ZEB1 axis can be inhibited by onco-suppressor miRs; thirdly, miRs affect both expression of Wnt and nuclear translocation of β-catenin; and finally, lncRNAs can regulate miR/Wnt/ZEB1 axis. The mediation of the miR/ZEB1 axis by lncRNAs will be more extensively discussed in the next section.

#### 4.1.2. LncRNAs as Modulators of miR/ZEB1 Axis

Long non-coding RNAs (lncRNAs) belong to a category of ncRNAs with regulatory effect on biological events [121,122]. They consist of at least 200 nucleotides and they are able to function as upstream mediators of miRs [111]. LncRNAs suppress the expression of miRs via acting as competitive endogenous RNA (ceRNA) [123]. The effect of lncRNAs on miR/ZEB1 axis has been investigated in cancer cells. For instance, miR-429 was found to inhibit EMT through ZEB1 inhibition and its expression is typically down regulated in pancreatic cancer cells. MiR-429 can be regulated by lncRNA XIST, which is up-regulated in pancreatic cancer cells to reduce miR-429 levels. This in turn increases ZEB1 expression and promotes EMT. Additionally, through targeting the miR-429/ZEB1 axis, XIST also affects morphology of cancer cells, such that silencing XIST results in a change in cell morphology, from the original spindle shape to a rounded one [124]. In another instance, LncRNA IUR is a onco-suppressor factor that has shown a great capability in suppressing tumorigenesis [125]. LncRNA IUR can inhibit the migration and metastasis of prostate cancer cells via enhancing the expression of miR-200, which in turn inhibits ZEB1 [126].

Conversely, lncRNA TDRG1 is an oncogenic factor that is able to regulate miRs in cancer cells [127,128]. In lung cancer cells, TDRG1 enhances the migration, metastasis, and malignancy of cancer cells by promoting ZEB1 expression through miR-873-5p down-regulation [129]. LncRNA TTN-AS1 is also considered an oncogenic factor that induces ZEB1 through miR-4677-3p down-regulation, leading to the enhanced migration and metastasis of NSCLC cells [130]. LncRNA (Nuclear Enriched Abundant Transcript 1) NEAT1 contributes to enhancing the malignancy of cancer cells [131]. It has been demonstrated that NEAT1 can target miRs to regulate cancer proliferation and migration [132]. In breast cancer cells, NEAT1 reduces the expression of miR-448 to elevate the metastasis and invasion of cancer cells through ZEB1 up-regulation [133]. LncRNA TP73-AS1 reduces the expression of miR-200a to up-regulate ZEB1, leading to the enhanced progression and malignancy of tumor cells. There appears to be a feedback loop, wherein TP73-AS1-activated ZEB1 has a stimulatory effect on the expression of TP73-AS1 to enhance its inhibitory activity on miR-200a, leading to increased induction of ZEB1 [90].

In renal cell carcinoma (RCC), miR-429 typically reduces the expression of ZEB1 to suppress RCC progression. However, miR-429 can be inhibited by SCAMP1, a lncRNA that is activated by oxidative stress [134]. This highlights that stimulation of oxidative stress negatively impacts cancer therapy. Generally, it is believed that enhancing level of oxidative stress can lead to a reduction in the viability of cancer cells by predisposing them into apoptosis [135,136]. However, as mentioned, increasing levels of oxidative stress may also activate lncRNAs involved in cancer metastasis. Therefore, careful considerations are warranted before using oxidative stress in cancer therapy, keeping in mind the possible adverse effects of this treatment method.

MiR-139-5 was also found to suppress ZEB1 levels. However, lncRNA human leukocyte antigen (HLA) complex 5 (HCP5) is able to induce ZEB1 and EMT by suppressing miR-139-5 [137]. LncRNA MAGI2-AS3 has also been explored in cancer and it appears that MAGI2-AS3 is able to modulate molecular pathways such as Fas/FasL to suppress breast cancer, bladder cancer, and hepatocellular carcinoma [138,139]. Particularly, in gastric cancer cells, miR-141/200a diminishes the invasion and migration of tumor cells via suppressing ZEB1. LncRNA MAGI2-AS3 down-regulates the expression of miR-141/200a to induce ZEB1, leading to the stimulation of EMT and enhanced invasion of tumor cells [125].

LncRNA LINC00511 is located on chromosome 17q24.3 and has been associated with increased malignancy in cancer [140]. In glioblastoma (GBM) cells, miR-524-5p inhibits ZEB1 to suppress GBM invasion and migration. LINC00511 has been found to decrease the expression of miR-524-5p to up-regulate YB1 [141]. YB1 is a transcription factor that can enhance the expression of ZEB1 in cancer [128]. The inhibition of miR-524-5p by LINC00511 promotes ZEB1 expression through YB1 up-regulation, leading to enhanced EMT and malignancy of GBM cells [141]. These studies again demonstrate that lncRNAs can disrupt inhibitory effects of miRs on ZEB1 to promote metastasis of cancer cells. In glioma cells, miR-205-3p inhibits TGF-β, while lncRNA linc00645 functions as an upstream mediator and activates TGF-β via suppressing miR-205-3p, leading to an increase in ZEB1 levels and subsequent EMT activation [137].

LncRNA MALAT1 located on the chromosome 11q13, is also suggested to be involved in elevating the malignancy of cancer cells. A variety of factors act as down-stream mediators for lncRNA MALAT1 and it appears that MALAT1 is capable of targeting miRs in cancer cells [105,142,143]. MALAT1 was found to enhance the expression of ZEB1 through miR-143-3p down-regulation, resulting in elevated migration and metastasis of tumor cells [144]. Another downstream target of MALAT1 is miR-429, which is considered as a potential biomarker for diagnosis of different cancers [145]. MALAT1 was found to inhibit miR-429 to accelerate the malignancy and invasion of cervical cancer cells [146]. Notably, miR-429 can inhibit the metastasis of cancer cells and stimulate apoptotic cell death through ZEB1 down-regulation [110]. Interestingly, it has been demonstrated that fine particulate matter (PM_2.5_, aerodynamic diameter, 2.5 μm) is able to induce oxidative stress, inflammation, genetic mutations, and DNA damage [147,148]. It has been found that miR-204 can reduce the expression of ZEB1 to suppress EMT. PM_2.5_ activates MALAT1 via stimulation of NF-κB, as an inflammatory pathway. MALAT1 in turn induces ZEB1 through miR-204 down-regulation to enhance the malignancy and invasion of tumor cells via EMT induction [149]. These two studies demonstrate that lncRNAs can affect more than one downstream miR to mediate ZEB1 levels, and that other molecular pathways such as NF-κB can act as upstream mediator of lncRNA/miR/ZEB1 axis.

HOXA distal transcript antisense RNA (HOTTIP) is located at the distal end of HOXA gene cluster [150]. This lncRNA undergoes abnormal expressions in different cancers and it has been shown that HOTTIP is related to the increased proliferation and progression of cancer cells [120]. It is held that lncRNA HOTTIP down-regulates the expression of miR-101 to elevate ZEB1 levels, leading to an increase in EMT [151]. A study has also shown that miR-205 down-regulates the expression of ZEB proteins and HOXD9 to suppress the malignancy and invasion of cancer cells through EMT inhibition [152]. Finally, it has been demonstrated that lncRNA HOXC-AS2 induces ZEB1 by sponging miR-876-5p, leading to the stimulation of EMT and enhanced migration and invasion of tumor cells [153]. Taken together, the relationship between lncRNAs and miRs in the regulation of ZEB1 in cancer cells are dynamic and complicated, and understanding these pathways is an essential part of effective cancer therapy.

#### 4.1.3. CircRNAs as Modulators of miR/ZEB1 Axis

Circular RNAs (circRNAs) are endogenous, conserved ncRNAs that are sometimes employed as biomarkers for cancer diagnosis [154,155]. Similar to lncRNA, circRNAs are able to modulate the expression of their targets [156]. In lung cancer cells, hsa-circ-0023404 decreases the expression of miR-217 to enhance the expression of its target, ZEB1, leading to the increased migration and invasion of cancer cells [157]. The laryngeal carcinoma is considered as one of the common cancers among head and neck tumors and is mainly diagnosed in elder people [158]. In spite of the low incidence rate, this cancer results in high mortality worldwide [159]. It was discovered that miR-200c is capable of inhibiting ZEB1 to prevent the metastasis and invasion of laryngeal cancer cells. Hsa-circ-005748 up-regulates ZEB1 by sponging miR-200c, leading to the metastasis of these cancer cells [160]. Therefore, inhibition of hsa-circ-005748 may in turn increase miR-200c expression to suppress ZEB1 and cancer metastasis. Similarly, in lung adenocarcinoma (LUAD) cells, miR-665 is able to inhibit cancer metastasis via ZEB1 down-regulation. The circ-TSPAN4 enhances the expression of ZEB1 by miR-665 down-regulation to promote the metastasis of LUAD cells [161]. These studies concur that down-regulation of onco-suppressor miRs in cancer cells may also be mediated by upstream circRNAs. This in turn promotes up-regulation of ZEB1 and enhanced metastasis of cancer cells.

### 4.2. ZEB2

#### 4.2.1. MiRs as Modulators of ZEB2

MiR-124 is suggested to be an onco-suppressor miR. Recently, an effort has been made to suppress the prostate cancer invasion. It is held that cationic polymer nanoparticles are able to deliver miR-124 in prostate cancer cells to inhibit their proliferation, motility, and colony formation [162]. In TNBC cells, miR-124 effectively decreases the malignancy and invasion of tumor cells by EMT inhibition through ZEB2 down-regulation [163]. Similarly, miR-145 has been widely established as a tumor suppressor. It negatively affects the invasion and migration of thyroid carcinoma cells by down-regulation of NF-κB signaling pathway [164]. Furthermore, lncRNA-ROR down-regulates the expression of miR-145 to remove its inhibitory impact and induce EMT in tumor cells [165]. Importantly, it was found that miR-145 decreases the expression of ZEB2 to inhibit EMT, and consequently, suppress the proliferation, progression, and migration of NSCLC cells [166]. These studies demonstrate that the downregulation of ZEB2 by onco-suppressor miRs can lead to a decrease in the metastasis of cancer cells.

Another onco-suppressor miR is miR-30a. In breast cancer cells, miR-30a suppresses the nuclear translocation of β-catenin to attenuate cancer proliferation and progression, and is associated with favorable prognosis of patients with breast cancer [167]. Furthermore, miR-30a appears to be beneficial in sensitizing cancer cells to chemotherapy via affecting Akt signaling pathway [168]. MiR-30a was found to inhibit ZEB2 to result in a reduction of triple negative breast cancer (TNBC) cells malignancy [169]. Finally, miR-3653 is an onco-suppressor that is down-regulated in hepatocellular carcinoma (HCC) cells [170]. MiR-3653 was found to bind to the 3′-UTR of ZEB2 to diminish its expression, leading to the reduced invasion and malignancy of colon cancer cells [171]. MiR-138-5p uses a same strategy in inhibition of lung adenocarcinoma cell malignancy, by suppressing EMT through ZEB2 inhibition to attenuate metastasis of tumor cells [172].

Osteosarcoma typically has a high recurrence rate and low survival rate [173,174]. Therefore, understanding the pathways involved in malignancy and cancer progression may pave the road for improved treatment of this type of cancer. Investigation of molecular pathways has shown that miR-101 up-regulation inhibits ZEB2 and affects proliferation and metastasis of osteosarcoma cells [175]. Unfortunately, miR-101 is down-regulated in osteosarcoma cells compared to the normal cells. Enhancing the expression of miR-101 may reduce malignancy and progression of osteosarcoma cells.

#### 4.2.2. LncRNAs as Modulators of miR/ZEB2 Axis

In the previous section, we demonstrated that lncRNAs are able to function as ceRNA in affecting miR expression. Notably, increasing evidence has also demonstrated that lncRNAs can effectively target ZEB2 via affecting miRs. For instance, LncRNA HOTAIRM1, which has dual properties as it interacts with both onco-suppressor and oncogenic miRs. HOTAIRM1 is located on human HOXA gene cluster and suggested to be involved in myeloid cell development [176]. A newly published article has shown the anti-tumor activity of lncRNA HOTAIRM1 by up-regulation of ARHGAP24 through miR-106a-5p inhibition [177]. However, it has been found that HOTAIRM1 is related to the elevated migration and invasion of tumor cells [178]. It is held that lncRNA HOTAIRM1 diminishes the expression of miR-873-5p to induce ZEB2, resulting in an increase in cancer cell proliferation and suppressing apoptotic cell death [179].

MiR-505 is also considered an onco-suppressor miR that interacts with IGF-1 and HMGB1 to suppress the growth and malignancy of tumor cells [180,181,182]. Various studies have demonstrated that lncRNAs such as lncRNA CRAL, LEF-AS1, and DLX6-AS1 are able to target miR-505 in different cancers such as gastric cancer, CRC, and breast cancer [183,184,185]. In cervical cancer, lncRNA CTS was found to target miR-505. MiR-505 down-regulates ZEB2 levels to inhibit EMT and invasion of cervical cancer cells. LncRNA CTS, therefore, stimulates ZEB2-mediated EMT through miR-505 sponging, leading to the enhanced viability, proliferation, and malignancy of cervical cancer cells [186]. Taken together, stimulation of ZEB2 by lncRNAs not only enhances metastasis of cancer cells via EMT induction, but also promotes cell proliferation. This decrease in apoptosis by ZEB2 induction is of importance in chemotherapy, since cancer cells can attain chemoresistance via reducing their sensitivity into chemotherapy-mediated apoptosis. As such, targeting miR/ZEB2 axis may be a promising strategy in cancer therapy, as it increases the sensitivity of cancer cells toward chemotherapy.

In gastric cancer cells, miR-203 diminishes cancer metastasis through ZEB2 down-regulation. LncRNA UCA1 enhances the progression and metastasis of tumor cells through disrupting the miR-203/ZEB2 axis [187]. Particularly, lncRNAs can affect upstream transcription factors of ZEB2 in cancer metastasis. In NSCLC cells, slug was found to behave as an upstream mediator to induce EMT through increasing ZEB2 levels. MiR-218 was able to disrupt the Slug/ZEB2 axis to suppress NSCLC migration. Conversely, miR-218 undergoes down-regulation by lncRNA SNHG12 to stimulate Slug/ZEB2 and promote metastasis of NSCLC cells [188].

Glioma is an intracranial tumor that emanates from neuroglial stem or progenitor cells [189]. Again, this is an alarming cancer with high mortality and morbidity rate [190,191]. The migration and invasion of cancer cells into neighboring cells and tissues reduces the survival time of patients [192,193]. It has been demonstrated that lncRNA SNHG5 can inhibit miR-205-5p expression. Reduced miR-205-5p expression triggers the induction of ZEB2, which in turn enhances the migration ability of tumor cells [194]. Up-regulation of miR-205-5p may therefore be beneficial in reducing glioma malignancy.

#### 4.2.3. CircRNAs as Modulators of miR/ZEB2 Axis

Increasing evidence highlights the role of miR-377 as an onco-suppressor in cancer cells. MiR-377 can target Akt signaling to suppress the proliferation and invasion of tumor cells, and induce cell cycle arrest [195]. Normally, miR-377 reduces the expression of ZEB2. In bladder cancer cells, the expression of miR-377 undergoes down-regulation by circZFR to promote cancer metastasis through ZEB2 stimulation [196]. MiR-653 also appears to be an onco-suppressor miR in bladder cancer cells. CircRNA ciRs-6 reduces miR-653 expression to induce March1, leading to the increased proliferation of tumor cells [110]. MiR-653 is similarly suppressed in breast cancer cells, by another circRNA hsa-circ-0004771. Knockdown of hsa-circ-0004771 sensitizes cancer cells to apoptosis and inhibits their progression through miR-653 up-regulation and subsequent inhibition of ZEB2 [197]. Evidently, ZEB2 induction dually enhances proliferation and metastasis of cancer cells. Hence, targeting the circRNA/miR/ZEB2 axis can pave the way into effective inhibition of proliferation and migration of cancer cells.

In renal cancer, patients typically have poorer survival rates and treatment strategies can be improved [198,199,200]. MiR-153 was found to exert inhibitory impact on ZEB2 expression to suppress renal cancer, while circPCNXL2 stimulates ZEB2 expression via miR-153 sponging to elevate the invasion and proliferation of renal cancer cells [201]. Therefore, decreasing the expression of circPCNXL2 may yield an up-regulation of miR-153 and suppresses ZEB2 expression to eliminate renal cancer.

**Figure 1:**
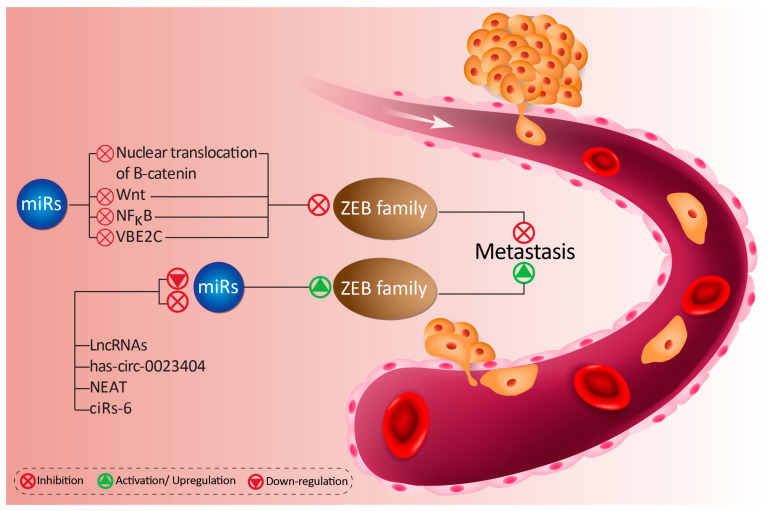
The oncogenic upstream mediators of miRs activating ZEB proteins, which enhances metastasis and migration of cancer cells.

**Figure 2:**
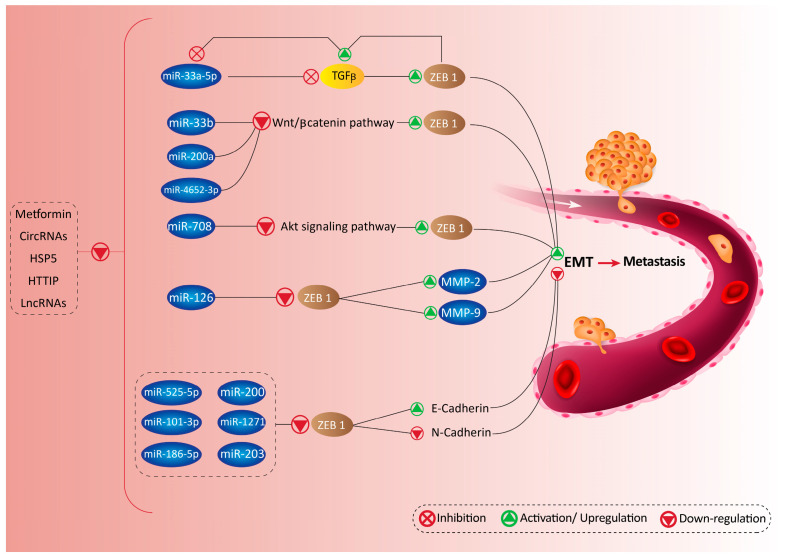
Metformin, lncRNAs, circRNAs, and other molecular pathways are able to function as upstream mediators of miRs in targeting ZEB proteins, and promote cancer progression.

In all, these studies highlight the extensive influence that miRs have on ZEB proteins (Figure 1 and Figure 2). Across a wide range of cancers, different miRs work to inhibit ZEB and halt cancer progression. Additionally, lncRNAs and circRNAs are able to act as upstream mediators of miRs to affect ZEB2 expression. Through the revealing of these molecular pathways, we may better understand these promising candidates in cancer therapy.

## 5. MicroRNAs, ZEB, and Their Role in Tumor Resistance

Multidrug resistance (MDR) is a complicated and challenging phenomenon accounting for cross-resistance towards structurally unrelated drugs [202,203]. It is estimated that approximately 70% of solid and hematological tumors demonstrate MDR. This percentage elevates after chemotherapy, since cancer cells are able to switch among molecular pathways to obtain chemoresistance, and frequent application of chemotherapeutic agents speeds up MDR [204,205]. Therefore, when trying to understand the role of miR and ZEB proteins in cancer, it also important to explore how miR’s modulation on ZEB can affect tumor resistance. Particularly, miR is implicated in tumor resistance. For instance, cisplatin is a potential chemotherapeutic agent with the ability of inhibiting the proliferation and viability of various cancers [206]. In ovarian cancer, it has been reported that miR-137 reduces the expression of MCL1 to sensitize tumor cells into cisplatin-induced apoptosis [207]. In this section, we seek to understand how miR modulation on ZEB can contribute to tumor resistance.

### 5.1. ZEB1

#### 5.1.1. Paclitaxel Resistance

Paclitaxel (PTX) is a chemotherapeutic agent that is frequently employed in cancer therapy to prevent cell proliferation due to its anti-mitotic capabilities [208]. Unfortunately, PTX resistance is an important obstacle, which has reduced the feasibility of this agent [209,210,211,212]. Notably, ZEB1 can promote cancer cells resistance towards PTX, and down-regulation of ZEB1 may be a key toward re-sensitizing cancer cells to PTX chemotherapy [213]. MiR-124-3p suppresses ZEB1 to sensitize gastric cancer cells into PTX therapy. Circular RNA Circ-PVT1 reverses this axis by sponging miR-124-3p and elevating the expression of ZEB1 to induce PTX resistance in gastric cancer cells [214]. LncRNA NEAT1 was also found to mediate PTX resistance in ovarian cancer cells. Normally, miR-194 undergoes up-regulation to inhibit ZEB1 and subsequently, reduce the malignancy and invasion of cancer cells. LncRNA NEAT1 suppresses the inhibitory effect of miR-194 on ZEB1 to induce the resistance of ovarian cancer cells into PTX chemotherapy [215].

#### 5.1.2. Gemcitabine Resistance

Gemcitabine is a chemotherapeutic agent isolated from deoxycytidine, which is frequently applied in the treatment of breast cancer [216]. Gemcitabine triggers cell cycle arrest by binding into DNA or suppressing ribonucleotide reductase [217,218]. It appears that ZEB1 contributes to the gemcitabine resistance in TNBC cells. This study found that ZEB1 associates with Yes associated protein (YAP) to enhance cancer progression and proliferation and induces chemoresistance. Importantly, ZEB1 was found to be a target of miR-873, and that increasing miR-873 expression down-regulates the expression of YAP and ZEB1, and sensitizes tumor cells into gemcitabine therapy [219].

#### 5.1.3. Cisplatin Resistance

Another important factor to consider when exploring acquired tumor resistance is lncRNA, which can regulate miR, to in turn affect ZEB levels. Prostate cancer-associated transcription 1 (PCAT1) undergoes up-regulation in cancer cells to suppress cell death [220]. In gastric cancer cells, PCAT-1 induces the resistance of cancer cells into cisplatin therapy by stimulation of ZEB1 through miR-128 inhibition, leading to the enhanced progression and malignancy of gastric cancer cells [221]. Therefore, targeting the miR/ZEB1 axis may alleviate cisplatin resistance.

#### 5.1.4. 5-Fluorouracil

**Figure 3:**
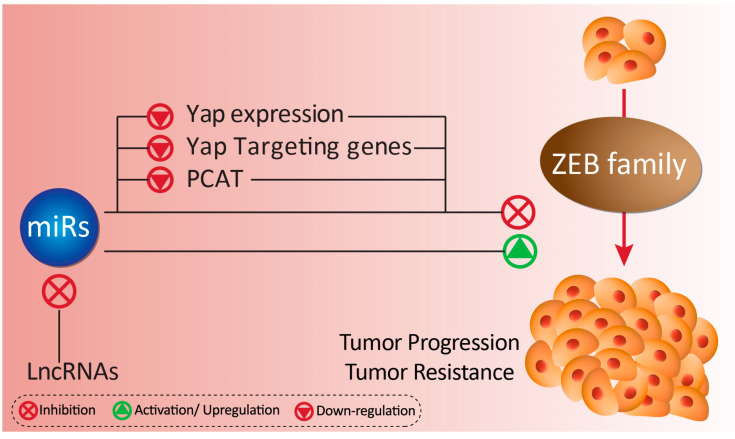
The miRs as key player in regulation of tumor malignancy via targeting ZEB proteins.

The most common chemotherapeutic agent in treatment of cancer is 5-FU [222]. Different molecular pathways are involved in resistance into 5-FU, and miRs are key players [223,224]. LncRNA NEAT1 have been found to possess oncogenic activity and enhance the progression and malignancy of cancer cells via targeting miRs such as miR-144-3p and miR-410 [225,226]. In CRC cells, NEAT1 is involved in 5-FU resistance through miR-34a regulation [227] (Figure 3).

### 5.2. ZEB2

In osteosarcoma, miR-200b diminishes the progression and motility of tumor cells by inhibition of PI3K/Akt and AMPK signaling pathways, leading to the downregulation of vascular endothelial growth factor (VEGF). LncRNA CCAT2 reverses this axis by induction of VEGF through miR-200b inhibition [228]. It is worth mentioning that enhancing the expression of miR-200b is beneficial in sensitizing cancer cells into chemotherapy, so that arrestin domain containing 3 (ARRDC3) elevates the efficacy of chemotherapy in TNBC cells via miR-200b up-regulation [229]. It appears that up-regulation of miR-200b enhances apoptosis in lung cancer cells and remarkably increases the efficacy of chemotherapy [230]. Another member of miR-200 family, known as miR-200c, sensitizes gastric cancer cells to cisplatin and enhances chemotherapeutic efficacy by suppressing ZEB2 expression [215].

## 6. MicroRNAs Target ZEB Family in Immune Cells

Other than ZEB’s prevalent role in EMT, ZEB’s involvement with the tumor microenvironment and immune system is also crucial in its mediation of cancer dissemination and development. Tumor cells use immunosuppressive cells such as CD4+ T cells to escape from the anti-cancer activity of CD8+ T cells [231,232,233]. Notably, it has been demonstrated that cytotoxic CD8+ tumor infiltrating lymphocytes (CD8+ TILs) are able to eliminate cancer cells [234], while sustained exposure of tumor cells into CD8+ TILs reduces their anti-tumor activity [235]. It is worth mentioning that PD-1/PD-L1 axis may be involved in driving CD8+ T cell exhaustion and therapies targeting PD-L1 have been explored [236,237,238,239,240]. PD-L1 binds to PD-1 to induce apoptotic cell death in CD8+ T cells and ensure the survival of cancer cells [241,242,243,244].

In diffuse large B cell lymphoma (DLBCL) cells, miR-8890-3p is capable of suppressing ZEB1, while lncRNA SNHG14 conversely reduces the expression of miR-8890-3p to activate ZEB1. Consequently, ZEB1 stimulates PD-L1 to protect cancer cells against the cytotoxic effects of immune cells, resulting in promoting the survival and migration of DLBCL cells [245].

**Figure 4:**
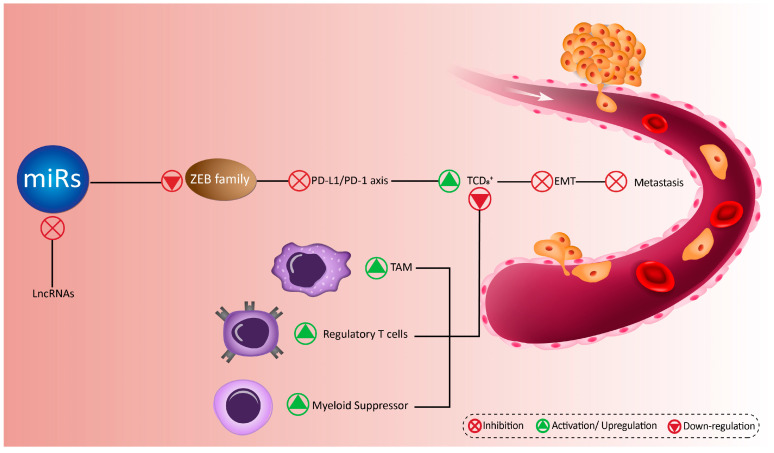
How tumor microenvironment components are affected by the relationship between miRs and ZEB proteins, and their regulation by lncRNAs.

In another instance, it has been reported that ZEB1 is an efficient factor in elevating the malignancy of tumor cells, through the induction of PD-L1 expression to enhance the levels of CD8+ T-cell immunosuppression and cancer metastasis. Enhancing the expression of miR-200 disrupts ZEB1 expression to suppress PD-L1 and immunosuppression, resulting in decreased metastasis and invasion of cancer cells [246]. ZEB1 can induce EMT in breast cancer cells via activation of PD-L1. It has been reported that miR-200 overexpression reduces the levels of ZEB1 to inhibit EMT through interfering with PD-L1 activation, as an immunosuppressive factor [247]. Unfortunately, there are currently no reports about the relationship between miRs and ZEB2 in cancer immunotherapy, and further studies can focus on revealing relationship between miR/ZEB2 axis and cancer immunotherapy. Table 1, Table 2, Table 3, Table 4, Table 5 and Table 6 demonstrate the regulation of ZEB1 and ZEB2 by various miRs proteins in mediating cancer metastasis. Upstream mediators of miR such as lncRNAs and circRNAs are also highlighted in Table 1 through Table 6. Figure 4 further summarizes the effect of miR/ZEB axis on immune system.

## 7. Conclusions

In this article, we provided a comprehensive review about the relationship between miRs and ZEB family in cancer cells and how this relationship affects the progression and metastasis of tumor cells. After miRs discovery, an exponential amount of research has been performed to understand their role in different biological processes such as cell differentiation, apoptosis, and migration. We typically observe aberrant miR expression in cancer cells and restoring the normal expression of miRs may be crucial in cancer therapy. It is also vital to explore the relevance of ZEB1 and ZEB2 proteins in cancer therapy. It has been reported that ZEB proteins are able to enhance the proliferation and malignancy of tumor cells. One of the most important pathways affected by ZEB proteins is the EMT mechanism. It appears that induction of EMT by ZEB proteins not only enhances the progression and metastasis of cancer cells, but also stimulates drug resistance. Therefore, revealing the underlying molecular pathways involved in ZEB regulation can be beneficial for further studies in the field of cancer therapy and elevating the efficacy of chemotherapy. In this review, we also detailed how and which miRs affect ZEB proteins in various cancers. We consolidated the factors that may function as upstream modulators to negatively affect miRs, leading to the induction of ZEB expression. As it is shown in Table 1, Table 2, Table 3, Table 4, Table 5 and Table 6, lncRNAs and circRNAs can act as oncogenic factors. These upstream mediators induce and enhance the expression of ZEB1 and -2 through sponging their target miRs, resulting in an increase in malignancy and invasion of tumor cells. Identification of these factors and further targeting of them can significantly diminish the malignancy of tumor cells and pave the road for the effective cancer therapy. Finally, we highlighted ZEB1’s role in immunosuppression. Through it, we identified a crucial knowledge gap wherein the relationship between miRs, ZEB2, and immune cells in the cancer context is still a mystery. In all, we dissected the different effects of miR on ZEB proteins, which may in turn help us develop better treatment strategies in attenuating metastasis of cancer cells.

## Figures and Tables

**Table 1:** ZEB1 regulation by miRs in different cancers.

MiR	Down-Stream Target	Cancer Type	Major Outcomes	Refs
**MiR-23a**	ZEB1	Intraocular tumor	A negative feedback loop between miR-23a and ZEB1 regulates EMT and overexpression of miR-23a inhibits EMT by ZEB1 down-regulation	[248]
**MiR-23b**	ZEB1	Bladder cancer	MiR-23b induces apoptosis and cell cycle arrest, and decreases the invasion and EMT through ZEB1 inhibition	[249]
**MiR-33b**	ZEB1	Melanoma	Cordycepin enhances the expression of miR-33b to inhibit ZEB1 and induces mesenchymal-epithelial transition in cancer cells, resulting in decreased invasion and migration of cancer cells	[250]
**MiR-126**	ZEB1	Osteosarcoma	Inhibition of EMT, migration, and metastasis of cancer cells through ZEB1 down-regulation	[251]
**MiR-128**	ZEB1	Prostate cancer	MiR-128 sensitizes cancer cells into cisplatin chemotherapy by ZEB1 down-regulation and decreasing the malignancy and invasion of cancer cells	[252]
**MiR-130b**	ZEB1	Endometrial cancer	The miR-130b down-regulates the expression of ZEB1 to inhibit the malignancy and invasion of cancer cells	[253]
**MiR-139-5p**	ZEB1/2	Hepatocellular carcinoma	Reduced invasion, migration, metastasis, and EMT by ZEB1/2 down-regulation through miR-139-5p	[254]
		Glioblastoma multiforme	Suppressing the invasion and migration of cancer cells through ZEB1/2 inhibition	[255]
**MiR-141 and miR-146b-5p**	AUF1/ZEB1	Osteosarcoma	These miRs are able to down-regulate the expression of AUF1 to repress ZEB1, resulting in an increase in epithelial markers (E-cadherin and Epcam) and a decrease in mesenchymal markers (N-cadherin and Vimentin)	[256]
**MiR-144** **MiR-144**	ZEB1/2	Breast cancer	MiR-144 is an onco-suppressor that inhibits EMT and migration invasion through ZEB1/2 down-regulation	[257]
		Thyroid cancer	MiR-144 down-regulates the expression of ZEB1/2 to prevent cancer progression and proliferation	[258]
**MiR-150**	ZEB1	Esophageal squamous cell carcinoma	MiR-150 degrades ZEB1 to induce mesenchymal-epithelial transition (MET), resulting in a decrease in tumor depth, lymph node metastasis, and lymphatic invasion	[259]
		Ovarian cancer	Suppressing the malignancy and invasion of cancer cells through ZEB1 inhibition	[260]
**MiR-199a-3p**	ZEB1	Melanoma	The administration of gambogic acid is associated with up-regulation of miR-199a-3p and subsequent inhibition of ZEB1 to suppress cancer progression both in vitro and in vivo	[261]
**MiR-199b**	ZEB1	Non-small cell lung cancer	Suppressing the proliferation, migration, and invasion of cancer cells through ZEB1 down-regulation	[262]
**MiR-200**	ZEB1-FAK/Src	Human lung cancer	The miR-200 up-regulation decreases the invasion and malignancy of cancer cells through enhancing ZEB1 expression and subsequent activation of FAK/Src	[263]
	ZEB1	Endometrial carcinoma	The expression of miR-200 undergoes down-regulation in endometrial carcinoma cells to induce ZEB1 and subsequently, EMT mechanism to elevate the invasion and malignancy of cancer cells	[264]
		Lymphoma	Generation of a less aggressive behavior by ZEB1 inhibition through miR-200	[265]
		Insulinoma mouse model	Overexpression of miR-200 is associated with ZEB1 inhibition and decreased migration and proliferation of cancer cells	[266]
**MiR-200b**	ZEB1	Osteosarcoma	Overexpression of miR-200b is associated with down-regulation of ZEB1 and decreased invasion and malignancy of cancer cells	[267]
		Human hepatocellular carcinoma	By down-regulation of ZEB1, miR-200b reduces the stemness of cancer cells	[268]
**MiR-200b and miR-141**	ZEB1	Non-small cell lung cancer	The overexpression of miR-200b and miR-141 is related to the inhibition of ZEB1 and sensitizing cancer cells into nintedanib	[269]
**MiR-200c**	ZEB1	Human colon cancer	Suppressing the invasion and migration of cancer cells through ZEB1 down-regulation	[270]
		Gastric carcinoma	Overexpression of miR-200c is related to the ZEB1 down-regulation and enhanced levels of E-cadherin protein	[271]
		Human bladder cancer	Administration of sulforaphane is associated with miR-200c induction and subsequently, inhibition of ZEB1 and malignancy of cancer cells	[272]
		Non-small cell lung carcinoma	The cyclamen pseudibericum extract up-regulates miR-200c to induce ZEB1 down-regulation, resulting in suppressing cancer progression and proliferation	[273]
		Non-small cell lung cancer	MiR-200c sensitizes cancer cells to the gefitinib-mediated apoptosis by down-regulation of ZEB1	[274]
		Lung cancer	MiR-200c sensitizes lung cancer cells into crizotinib chemotherapy by inhibition of ZEB1, and subsequently, EMT inhibition	[275]
**MiR-200c and miR-141**	ZEB1	Glioma cell	The miR-200c and -141 synergistically inhibit ZEB1 to prevent the malignancy and invasion of cancer cells	[276]
	ZEB1/2	Gastric cancer	MiR-200c/141 significantly decreases ZEB1/2 expression to suppress cancer malignancy	[277]
**MiR-203**	ZEB1	Non-small cell lung cancer	The administration of silymarin enhances the expression of miR-203 to inhibit ZEB1 and elevate the levels of E-cadherin, resulting in suppressing cancer	[278]
**MiR-204**	ZEB1	Prostate cancer	MiR-204 up-regulation sensitizes cancer cells into docetaxel-mediated apoptosis through ZEB1 down-regulation	[279]
**MiR-205**	ZEB1	Ovarian cancer	MiR-205 enhances the invasion and migration of cancer cells via ZEB1 up-regulation. Reducing the expression of miR-205 is of interest in suppressing the malignancy of cancer cells	[280]
		Prostate cancer	By inhibition of ZEB1, miR-205 sensitizes cancer cells into radiotherapy and induces DNA damage	[281]
		Breast cancer	MiR-205 sensitizes cancer cells into radiotherapy and prevents DNA repair by ZEB1 down-regulation	[282]
**MiR-205-5p**	ZEB1	Prostatic carcinoma	Suppressing the migration and invasion of cancer cells by ZEB1 down-regulation	[283]
**MiR-340**	ZEB1/TGF-β	Breast cancer	MiR-340 inhibits ZEB1 to suppress TGF-β-mediated cancer progression	[284]
	ZEB1	Osteosarcoma	MiR-340 down-regulates the expression of ZEB1 to sensitize cancer cells into cisplatin-mediated apoptotic cell death	[285]
**MiR-409-3p**	ZEB1	Breast cancer	MiR-409-3p binds to the 3′-UTR of ZEB1 to inhibit the progression and metastasis of cancer cells	[286]
**MiR-429**	ZEB1	Ovarian cancer	Down-regulation of miR-429 is related to the resistance of cancer cells into cisplatin chemotherapy. Up-regulation of miR-429 suppresses ZEB1 to sensitize cancer cells into apoptosis	[287]
		Oral squamous cell carcinoma	MiR-429 suppresses the viability and progression of cancer cells via ZEB1 down-regulation	[288]
		Human thyroid cancer	MiR-429 binds to the 3′-UTR to inhibit ZEB1, resulting in suppressing invasion of cancer cells	[110]
**MiR-431**	ZEB1	Hepatocellular carcinoma	MiR-431 suppresses the migration and invasion capabilities of cancer cells through inhibition of ZEB1-mediated EMT	[289]
**MiR-448**	ZEB1/2	Breast cancer	The miR-448 significantly reduces the expressions of ZEB1/2 to inhibit the malignancy and invasion of cancer cells via EMT down-regulation	[290]
**MiR-455**	ZEB1	Non-small cell lung cancer	The miR-455 reduces the expression of ZEB1 to inhibit the malignancy of cancer cells	[291]
**MiR-484**	Smad2/ZEB1	Cervical cancer	Overexpression of miR-484 inhibits Smad2/ZEB1 to suppress cancer malignancy and miR-484 expression can be considered as a biomarker	[292]
**MiR-508**	ZEB1	Renal cell carcinoma	Up-regulation of miR-508 significantly reduces the expression of ZEB1 to inhibit EMT, leading to a decrease in cancer migration and metastasis	[293]
**MiR-508-3p**	ZEB1	Triple negative breast cancer	Suppressing the invasion and EMT of cancer cells by down-regulation of ZEB1	[294]
**MiR-574-3p**	ZEB1	Human gastric carcinoma	MiR-574-3p reduces the expression of ZEB1 by binding into 3′-UTR to decrease the malignancy of cancer cells, and simultaneously, sensitize cancer cells into cisplatin therapy	[295]
**MiR-590-3p**	ZEB1/2	Glioblastoma multiforme	Decreased invasion and migration of cancer cells by ZEB1/2 down-regulation	[296]
**MiR-641**	ZEB1	Cervical cancer	Negatively affecting the proliferation, migration, and invasion of cancer cells through ZEB1 down-regulation	[297]
**MiR-652**	ZEB1	Pancreatic cancer	Acidic microenvironment of tumor cells induces EMT through ZEB1 up-regulation. Enhancing the expression of miR-652 inhibits acidic-mediated EMT and ZEB1 induction	[298]
**MiR-655**	TGF-β/ZEB1	Pancreatic cancer	MiR-655 inhibits TGF-β/ZEB1 axis to suppress EMT in cancer cells	[299]
**MiR-675-5p**	UBQLN1/ZEB1/miR200	Pancreatic cancer	The miR-675-5p reduces the malignancy of cancer cells and ZEB1 protein by up-regulation of UBQLN1 and down-regulation of miR-200	[300]
**MiR-873-5p**	ZEB1	Colorectal cancer	The inhibitory effect of miR-873-5p on the migration, EMT formation, and invasion of cancer cells is mediated through ZEB1 down-regulation	[301]
**MiR-875-5p**	EGFR/ZEB1	Prostate cancer	By suppressing EGFR/ZEB1 axis, miR-875-5p inhibits EMT mechanism and sensitizes cancer cells to radiotherapy	[302]
**MiR-1271**	ZEB1	Pancreatic cancer	Suppressing the invasion, progression, and EMT in cancer cells by ZEB1 down-regulation	[303]
**MiR-1236-3p**	ZEB1	High-grade serous ovarian carcinoma	There is a negative relationship between miR-1236-3p and ZEB1 to suppress the migration and invasion of cancer cells	[304]
**MiR-1236-3p**	ZEB1	Breast cancer	ZEB1 inhibition by miR-1236-3p contributes to the inhibitory effect of this miR on the migration and invasion of cancer cells	[305]
**MiR-3662**	ZEB1	Melanoma	Amelioration of invasiveness and malignancy of cancer cells by ZEB1 down-regulation	[306]

**Table 2:** miR/ZEB1 regulation by lncRNAs in different cancers.

LncRNA	MiR	Down-Stream Target	Cancer Type	Major Outcomes	Refs
**LncRNA DANCR**	MiR-33a-5p	ZEB1	Esophageal squamous cell carcinoma	MiR-33a-5p suppresses cancer malignancy via reducing ZEB1 expression. LncRNA DANCR sponges miR-33a-5p to enhances the invasion via ZEB1 induction	[307]
**LncRNA SNHG6**	MiR-101-3p	ZEB1	Hepatocellular carcinoma	LncRNA SNHG6 down-regulates the expression of miR-101-3p to induce ZEB1 and enhance the malignancy of cancer cells	[308]
**LncRNA PTAR**	MiR-101-3p	ZEB1	Serous ovarian cancer	LncRNA PTAR decreases the expression of miR-101-3p to induce ZEB1 and EMT mechanism, leading to the invasion and metastasis of cancer cells	[309]
**LncRNA NNT-AS1**	MiR-142-3p	ZEB1	Breast cancer	Enhancing the progression of cancer cells by sponging miR-142-3p and induction of ZEB1	[310]
**LncRNA TUG1**	MiR-142-3p	ZEB1	Hepatocellular carcinoma	By down-regulation of miR-142-3p, lncRNA TUG1 enhances the expression of ZEB1 to ensure the proliferation and malignancy of cancer cells	[311]
**LncRNA SNHG16**	MiR-140-5p	ZEB1	Esophageal squamous cell carcinoma	The lncRNA SNHG16 functions as an oncogenic factor and neutralizes the inhibitory effect of miR-140-5p on ZEB1 to induce EMT and enhance the migration and invasion of cancer cells	[312]
	MiR-205	ZEB1	Osteosarcoma	SNHG16 reduces the expression of miR-205 to elevate the expression of ZEB1, resulting in an increase in the viability, proliferation, and migration of cancer cells	[313]
**LncRNA HOTAIR**	MiR-217	ZEB1	Osteosarcoma	By reducing the expression of miR-217, lncRNA HOTAIR enhances the expression of ZEB1 and improves their malignancy	[314]
	MiR-23b-3p	ZEB1	Hepatocellular carcinoma	The miR-23b-3p inhibits ZEB1 and lncRNA HOTAIR prevents the inhibitory effect of miR-23b-3p on ZEB1 to induce EMT	[315]
**lncRNA UCA1**	Has-miR-145	ZEB1/2-FSCN1	Bladder cancer	There is a reverse relationship between lncRNA UCA1 and has-miR-145. Decreased expression of has-miR-145 enhances the expression of ZEB1/2 and FSCN1 to elevate the migration and invasion of cancer cells	[316]
	MiR-204-5p	ZEB1	Glioma cells	By sponging miR-204-5p, lncRNA UCA1 stimulates ZEB1 and activates EMT mechanism	[317]
**LncRNA ZEB1-AS1**	MiR-200c/141	ZEB1	Glioma cancer	LncRNA ZEB1-AS1 down-regulates the expression of miR-200c/141 to induce ZEB1 and enhance the malignancy and invasion of cancer cells	[318]
	MiR-409-3p	ZEB1	Non-small cell lung cancer	A feedback loop is involved, so that lncRNA ZEB1-AS1 induces ZEB1 through miR-409-3p down-regulation, leading to the metastasis and survival of cancer cells	[319]
	MiR-101	ZEB1	Colorectal cancer	Elevating the proliferation and migration of cancer cells via down-regulation of MiR-101 and up-regulation of ZEB1 by lncRNA ZEB1-AS1	[320]
**LncRNA MIAT**	MiR-150-5p	ZEB1	Osteosarcoma	The miR-150-5p is down-regulated by MIAT to induce ZEB1 and enhance the malignancy of cancer cells	[321]
**LncRNA MAGI1-IT1**	MiR-200a	ZEB1/2	Ovarian cancer	Via competitively binding into miR-200a, lncRNA MAGI1-IT1 enhances the expression of ZEB1/2 to ensure the invasion and metastasis of cancer cells	[293]
**LncRNA HULC**	MiR-200a-3p	ZEB1	Hepatocellular carcinoma	By sequestering miR-200a-3p, lncRNA HULC stimulates ZEB1 to enhance the malignancy and progression of tumor cells	[322]
**LncRNA NEAT1**	MiR-204	ZEB1	Nasopharyngeal carcinoma	MiR-204 inhibits EMT through ZEB1 down-regulation, and lncRNA NEAT1 reverse this axis to enhance the proliferation and viability of cancer cells	[323]
**LncRNA MINCR**	MiR-223	ZEB1-Akt/PI3K	Nasopharyngeal carcinoma	MINCR induces ZEB1 by sponging miR-223, resulting in activation of Akt/PI3K and resistance of cancer cells into radiotherapy	[324]
**LncRNA CAT104**	MiR-381	ZEB1	Gastric carcinoma	LncRNA CAT104 down-regulates the expression of miR-381 to enhances ZEB1 levels, resulting in enhanced invasion of cancer cells. Additionally, there is a negative feedback loop between ZEB1 and miR-381.	[325]
**LncRNA ZNF469-3**	MiR-574-5p	ZEB1	Triple negative breast cancer	The reverse relationship between ZNF469-3 and miR-574-5p paves the road for up-regulation of ZEB1 and subsequent activation of EMT, leading to the cancer progression and malignancy	[326]

**Table 3:** miR/ZEB1 regulation by various molecular pathways in different cancers.

Upstream Mediator	MiR	Down-Stream Target	Cancer Type	Major Outcomes	Refs
**ELF3**	MiR-141-3p	ZEB1	Hepatocellular carcinoma	Overexpression of miR-141-3p down-regulates ZEB1. The ELF3 reduces the expression of miR-141-3p to induce ZEB1 and EMT mechanism	[327]
**SPROUTY-2**	MiR-200/miR-150	ZEB1	Colon cancer	By reducing the expression of miR-200/miR-150, SPROUTY-2 induces ZEB1 to facilitate the mesenchymal phenotype acquisition of cancer cells	[286]
**STAT3**	MiR-200	ZEB1	Invasive breast carcinoma	ZEB1 stimulation by miR-200 down-regulation via STAT3-dependent manner enhances the EMT acquisition in cancer cells	[328]
**TGF-β1**	MiR-200	ZEB1/2	Non-small cell lung cancer	The administration of decitabine induces miR-200 expression through TGF-β1 inhibition to down-regulate ZEB1/2, leading to the suppressing EMT and migration of cancer cells	[329]
**GRHL2**	MiR-200b/a	ZEB1	Ovarian cancer	GRHL2 down-regulates the expression of ZEB1 by miR-200a/b overexpression to preserve the epithelial phenotype	[330]
**53BP1**	MiR-200b and miR-429	ZEB1	Breast cancer	The 53BP1 enhances the expression of miR-200b and miR-429 to elevate E-cadherin levels and suppress EMT mechanism through ZEB1 down-regulation	[331]
**Mel-18**	MiR-205	ZEB1/2	Breast cancer	Mel-18 enhances the expression of miR-205 to inhibit ZEB1/2, resulting in decreased progression and invasion of cancer cells	[332]
**ΔNp63α**	MiR-205	ZEB1	Cervical squamous cell carcinoma	ΔNp63α alleviates cancer progression and malignancy by enhancing the expression of miR-205, subsequently down-regulating of ZEB1, and consequently, inhibition of EMT, and enhancing E-cadherin levels	[333]
**KCNQ1OT1**	MiR-217	ZEB1	Colorectal cancer	KCNQ1OT1 inhibits miR-217 to stimulate ZEB1 and EMT mechanism in cancer cells. There is a feedback loop, so that ZEB1 also enhances the expression of KCNQ1OT1 to elevate its inhibitory effect on miR-217	[334]
**Circ008913**	MiR-889	DAB2IP/ZEB1	Skin carcinogenesis	Arsenite down-regulates the expression of circ008913 to up-regulate miR-889. Then, a decrease occurs in DAB2IP to induce ZEB1 and carcinogenesis	[335]
**Pituitary tumor-transforming gene 1**	MiR-3666	ZEB1	Cervical cancer	The expression of miR-3666 reduces to neutralize its inhibitory impact of ZEB1, and consequently, elevate the metastasis and progression of cancer cells	[289]

**Table 4:** ZEB2 regulation by miRs in different cancers.

MiR	Down-Stream Target	Cancer Type	Major Outcomes	Refs
**MiR-29b**	TET1/ZEB2	Breast cancer	The miR-29b is an oncogene miR that inhibits TET1 to induce ZEB2 expression, leading to the EMT and colony formation of cancer cells	[336]
**MiR-30a-5p**	ZEB2	Renal cancer	The miR-30a-5p reduces the expression of ZEB2 to be related with desirable prognosis of cancer cells	[337]
**MiR-101**	ZEB2	Osteosarcoma	Suppressing the invasion and proliferation of cancer cells through ZEB2 down-regulation	[175]
**MiR-124**	ZEB2	Triple negative breast cancer	MiR-124 diminishes the expression of ZEB2 to inhibit the EMT and invasion of cancer cells	[338]
**MiR-129**	Wnt-β-catenin/ZEB2	Non-small cell lung cancer	The miR-129 disrupts Wnt/ZEB2 axis to inhibit EMT	[339]
**MiR-132**	ZEB2	Colorectal cancer	Reducing the invasion and metastasis of cancer cells through ZEB2 down-regulation	[340]
		Lung cancer	Diminishing the migration and invasion of cancer cells through ZEB2 inhibition	[341]
**MiR-138**	ZEB2	Bladder cancer	The miR-138 binds to the 3′-UTR of ZEB2 to inhibit the metastasis and invasion of cancer cells	[289]
**MiR-141**	ZEB2	Hepatocellular carcinoma	The miR-141 decreases the expression of ZEB2 to induce apoptosis and diminish viability and proliferation of cancer cells	[342]
		Renal cancer	The administration of honokiol is associated with miR-141 induction and subsequent downregulation of ZEB2 to inhibit the malignancy of cancer cells	[343]
**MiR-145**	ZEB2	Non-small cell lung cancer	MiR-145 acts as an onco-suppressor miR that negatively affects the expression of ZEB2 to inhibit the progression and malignancy of cancer cells	[166]
		Prostate cancer	There is a negative feedback loop between miR-145 and ZEB2, so that overexpression of miR-145 down-regulates the expression of ZEB2 to ensure the reduced viability and proliferation of cancer cells	[344]
**MiR-145-5p**	ZEB2	Gastric cancer	The miR-145-5p decreases the levels of N-cadherin by ZEB2 down-regulation	[345]
**MiR-153**	ZEB2	Ovarian cancer	Acting as an onco-suppressor miR and reduces ZEB2 expression to EMT inhibition	[346]
**MiR-154**	ZEB2	Non-small cell lung cancer	The miR-154 exerts an anti-tumor impact by ZEB2 down-regulation	[347]
		Hepatocellular carcinoma	The miR-154 functions as an onco-suppressor miR by inhibition ZEB2 expression and reducing cancer malignancy and proliferation	[348]
**MiR-155 and FOXP3**	ZEB2	Colorectal cancer	The miR-155 and FOXP3 inhibit ZEB2 expression to suppress EMT via E-cadherin level up-regulation and Vimentin level downregulation	[307]
**MiR-187**	ZEB2	Osteosarcoma	The miR-187 decreases the expression of ZEB2 to inhibit the malignancy and migration of tumor cells	[349]
**MiR-200**	ZEB1/2	Ovarian cancer	The cancer cells acquire an epithelial phenotype by enhancing the expression of miR-200 and subsequent inhibition of ZEB1 and ZEB2 proteins	[350]
	ZEB2	Breast cancer	As an onco-suppressor miR, miR-200 decreases the expression of ZEB2 and its targets gene Snail1 to induce mesenchymal to epithelial transition	[351]
**MiR-200a**	ZEB2	Nasopharyngeal carcinoma	Suppressing the growth and invasion of cancer cells through ZEB2 down-regulation	[352]
		Hepatocellular carcinoma	The miR-200a diminishes the expression of ZEB2 to suppress EMT and invasion of cancer cells	[353]
		Ovarian cancer	The miR-200a increases the levels of E-cadherin by EMT inhibition and ZEB2 down-regulation	[354]
**MiR-200b**	ZEB2	Gastric carcinoma	Inhibition of ZEB2 by miR-200b suppresses invasion, metastasis, and migration of cancer cells	[355]
		Glioma	Reducing the growth and metastasis of ZEB2 inhibition	[356]
**MiR-200c**	ZEB2	Ovarian cancer	MiR-200c reduces the expression of ZEB2 to inhibit EMT by enhancing E-cadherin levels and reducing Vimentin levels	[357]
		Non-small cell lung cancer	The miR-200c inhibits EMT mechanism by ZEB2 down-regulation	[358]
**MiR-200c-3p**	ZEB2	Prostate carcinoma	The miR-200c-3p functions as an anti-tumor miR that inhibits the progression and invasion of cancer cells through ZEB2 down-regulation	[359]
**MiR-203**	ZEB2	Lung adenocarcinoma and nasopharyngeal carcinoma	MiR-203 enhances the efficacy of cisplatin in chemotherapy and eradication of cancer cells, and also inhibits their invasion by EMT down-regulation through ZEB2 inhibition	[360,361]
**MiR-205**	ZEB2	Renal cell carcinoma	The miR-205 is related to the favorable prognosis and reduced invasion of cancer cells through ZEB2 down-regulation	[362]
**MiR-206**	ZEB2	Renal cancer	Decreasing the proliferation of tumor cells through ZEB2 down-regulation	[363]
**MiR-211-5p**	ZEB2	Hepatocellular carcinoma	The miR-211-5p suppresses the metastasis of cancer cells via ZEB2 down-regulation	[215]
**MiR-215**	ZEB2	Non-small cell lung cancer	The in vitro and in vivo experiments demonstrate the potential of miR-215 in down-regulation of ZEB2 and suppressing the invasion, progression, and malignancy of cancer cells, and induction of apoptotic cell death	[364]
**MiR-335**	ZEB2	Colorectal cancer	The inhibition of metastasis and invasion of cancer cells through ZEB2 down-regulation	[365]
		Papillary thyroid cancer	Through reducing the expression of ZEB2, miR-335 suppresses the growth and metastasis of cancer cells	[366]
**MiR-338-3p**	ZEB2	Gastric cancer	MiR-338-3p diminishes the expression of ZEB2 to inhibit EMT in cancer cells	[367]
**MiR-454-3p and miR-374b-5p**	ZEB2	Bladder cancer	Reducing the expression of ZEB2 significantly decreases the migration and invasion of cancer cells	[325]
**MiR-506**	ZEB2	Gastric carcinoma	The miR-506 suppresses metastasis through ZEB2 down-regulation	[130]
**MiR-545**	Wnt-β-catenin/ZEB2	Non-small cell lung cancer	The miR-545 reduces the expression of Wnt/β−catenin to down-regulate the expression of ZEB2, leading to the decreased migration and invasion of cancer cells	[368]
**MiR-598**	ZEB2	Non-small cell lung cancer	The in vitro experiment demonstrated that miR-598 decreases the expression of ZEB2 to inhibit the migration and metastasis of cancer cells	[369]
**MiR-622**	ZEB2	Glioma	The increased expression of miR-622 is related to the desirable prognosis via ZEB2 down-regulation	[370]
**MiR-769-3p**	Wnt-β-catenin/ZEB2	Glioma	The miR-769-3p down-regulates the expression of Wnt and inhibits nuclear translocation of β−catenin to suppress ZEB2, leading to the decreased viability, proliferation and invasion of cancer cells	[371]
**MiR-940**	ZEB2	Glioma	Inhibition of cancer progression and EMT through ZEB2 down-regulation	[372]
**MiR-1179**	ZEB2	Hepatocellular carcinoma	The miR-1179 reduces the expression of ZEB2 to inhibit cancer progression and malignancy	[373]
**MiR-3653**	ZEB2	Colon cancer	Suppressing metastasis and EMT by inhibition of ZEB2	[171]

**Table 5:** MiR/ZEB2 regulation by lncRNAs in different cancers.

LncRNA	MiR	Down-Stream Target	Cancer Type	Major Outcomes	Refs
**LncRNA TUG1**	MiR-142	ZEB2	Bladder cancer	The lncRNA TUG1 stimulates ZEB2 through miR-142 down-regulation to inhibit apoptosis and enhance the proliferation of cancer cells	[374]
**LncRNA ROR**	MiR-145	ZEB2	Hepatocellular carcinoma	The lncRNA ROR elevates the expression of ZEB2 through miR-145 sponging to inhibit the EMT and malignancy of cancer cells	[375]
**LncRNA MALAT1**	MiR-200s	ZEB2	Kidney carcinoma	The lncRNA MALAT1 induces ZEB2 via miR-200s sponging, predisposing cancer cells into growth and proliferation	[376]
	MiR-204	ZEB2	Breast cancer	The negative relationship between MALAT1 and miR-204 results in ZEB2 induction to enhance the migration and invasion of cancer cells	[377]
**LncRNA UCA1**	MiR-203	ZEB2	Gastric cancer	This lncRNA sponges miR-203 to induce ZEB2, leading to the enhanced malignancy, invasion, and proliferation of tumor cells	[187]
**LncRNA SNHG5**	MiR-205-5p	ZEB2	Glioma	LncRNA SNHG5 stimulates ZEB2 by sponging miR-205-5p to elevate the proliferation of cancer cells	[194]
**LncRNA UICLM**	MiR-215	ZEB2	Colorectal cancer	The in vivo and in vitro experiments demonstrated that lncRNA induces ZEB2 via miR-215 down-regulation to enhance the migration and malignancy of cancer cells	[378]
**LncRNA SNHG12**	MiR-218	Slug/ZEB2	Non-small cell lung cancer	MiR-218 inhibits Slug/ZEB2 axis to suppress EMT in cancer cells. LncRNA SNHG16 activates Slug/ZEB2 axis through miR-218 sponging	[188]
**LncRNA XIST**	MiR-367 and miR-141	ZEB2	Non-small cell lung cancer	The lncRNA XIST up-regulates the expression of ZEB2 by inhibition of miR-367 and miR-141, leading to the TGF- β-induced EMT	[379]
**LncRNA UCA1**	MiR-498	ZEB2	Esophageal cancer	The lncRNA UCA1 inhibits ZEB2 via miR-498 down-regulation to suppress the migration, proliferation, invasion, and EMT	[7]
**LncRNA CTS**	MiR-505	ZEB2	Cervical cancer	Down-regulation of miR-505 by CTS is associated with increased malignancy of cancer cells through ZEB2 induction	[186]
**LncRNA HOTAIRM1**	MiR-873-5p	ZEB2	Glioma	LncRNA HOTAIRM1 decreases the expression of miR-873-5p by sponging to up-regulate the expression of ZEB2, leading to an increase in progression of glioma cells and a decrease in apoptotic cell death	[179]

**Table 6:** miR/ZEB2 regulation by various molecular pathways in different cancers.

Upstream Mediator	MiR	Down-Stream Target	Cancer Type	Major Outcomes	Refs
**P53**	MiR-30a	ZEB2	Breast cancer	P53 stimulates the expression of miR-30a to upregulate ZEB2, resulting in reduced viability, proliferation, and invasion of cancer cells	[169]
**EZH2-DNMT1**	MiR-142-3p	ZEB2	Nasopharyngeal carcinoma	The EZH2-DNMT1 induces ZEB2 through miR-142-3p sponging, resulting in an increase in cancer progression	[380]
**CircNUP214**	MiR-145	ZEB2	Thyroid cancer	CircNUP214 induces ZEB2 through miR-145 down-regulation to enhance the malignancy and progression of cancer cells	[381]
**CircPCNXL2**	MiR-153	ZEB2	Renal cancer	The circPCNXL2 stimulates the expression of ZEB2 through miR-153 down-regulation to suppress the malignancy and invasion of cancer cells	[201]
**FOXP3**	MiR-155	ZEB2	Human breast cancer	FOXP3 and miR-155 synergistically down-regulate the expression of ZEB2 to diminish the invasiveness of cancer cells	[382]
**Akt/ERK**	MiR-200c	ZEB2	Gastric cancer	The inhibition of Akt/ERK enhances the expression of miR-200c to suppress IGF-I-mediated ZEB2, leading to the reduced invasion and EMT of cancer cells	[383]
**β1 integrin**	TGF-β/miR-200	ZEB2	Triple negative breast cancer	Enhancing the expression of β1 integrin reduces the metastasis of cancer cells into lung. This is followed by disrupting TFG−β/miR-200/ZEB2, elevating the E-cadherin levels, and restoring the cohesion of cells	[384]
**CircZFR**	MiR-377	ZEB2	Bladder cancer	Enhanced progression and malignancy of cancer cells result from down-regulation of miR-377 by circZFR and subsequent induction of ZEB2	[196]
**Hsa-circ-0004771**	MiR-653	ZEB2	Breast cancer	MiR-653 reduces the expression of ZEB2 and is associated with desirable prognosis. Hsa-circ-0004771 diminishes miR-653 expression to induce ZEB2, leading to the inhibition of apoptosis and enhanced migration and invasion of cancer cells	[197]

## Abbreviations

EMT, epithelial-to-mesenchymal transition; PCD, programmed cell death; MET, mesenchymal-to-epithelial transition; TGF-β, transforming growth factor-beta; EMT-TFs, EMT-promoting transcription factors; ZEB, zinc finger E-box-binding homeobox; CZF, carboxy-terminal zinc finger cluster; CtBP, C-terminal binding protein; CRC, colorectal cancer; DAXX, death domain-associated protein; ROCK2, Rho associated coiled-coil containing protein kinase 2; NSCLC, non-small cell lung cancer; MTBP, MDM2 binding protein; IDO1, indoleamine-2,3-dioxygenase-1; MMP, matrix metalloproteinase; PI3K, phosphatidylinositol 3-kinase; Akt, protein kinase B; ncRNAs, non-coding RNAs; SnoRNAs, small nucleolar RNAs; 3′-UTR, 3′-untranslated region; mRNA, messenger RNA; pri-miR, primary miR; SIRT1, sirtuin 1; FOXO3a, Forkhead box O3; NOB1, nin one binding protein; PD-L1, programmed death ligand 1; OSCC, oral squamous cell carcinoma; mTOR, mammalian target of rapamycin; lncRNAs, long non-coding RNAs; ceRNA, competitive endogenous RNA; NEAT1, Nuclear Enriched Abundant Transcript 1; RCC, renal cell carcinoma; HLA, human leukocyte antigen; HCP5, HLA complex 5; HOTTIP, HOXA distal transcript antisense RNA; α-SMA, α-smooth muscle actin; circRNAs, circular RNAs; LUAD, lung adenocarcinoma; TNBC, triple negative breast cancer; HCC, hepatocellular carcinoma; MDR, multidrug resistance; P-gp, P-glycoprotein; PTX, paclitaxel; YAP, Yes-associated protein; PCAT1, prostate cancer-associated transcription 1; VEGF, vascular endothelial growth factor; ARRDC3, arrestin domain containing 3; CD8+ TILs, CD8+ tumor infiltrating lymphocytes; DLBCL, diffuse large B cell lymphoma.
